# Acute Abdomen in a Patient with Cancer Pain on Oxycodone

**DOI:** 10.1155/2011/858672

**Published:** 2011-10-31

**Authors:** Naomi Kishine, Atsunobu Tsunoda, Seiji Kishimoto, Tomohisa Shoko

**Affiliations:** ^1^Department of Otolaryngology, Tokyo Medical and Dental University, Tokyo 113-8519, Japan; ^2^Head and Neck Surgery, Tokyo Medical and Dental University, Tokyo 113-8519, Japan; ^3^ER Center, Tokyo Medical and Dental University, Tokyo 113-8519, Japan

## Abstract

Opioids are a mainstay of treatment for moderate to severe cancer pain. At present, oxycodone has fewer adverse effects compared to morphine and is widely used for cancer pain therapy. The adverse effects of oxycodone are similar to morphine and include constipation, nausea, and sedation. However, acute abdominal pain is rarely seen. Here, we describe a cancer patient presenting with acute abdomen with stercoral diarrhea. A 54-year-old man with squamous cell carcinoma of the external auditory canal had been taking oxycodone for pain relief. The patient had taken oxycodone for several months and had never complained of either diarrhea or constipation. After an increase in the dosage of oxycodone, he complained of abdominal distension and constipation. After being administered a laxative, he complained of diarrhea and severe abdominal pain. He visited the emergency department and was diagnosed with acute colonic obstruction caused by severe constipation. He self-medicated with oxycodone at dosages of up to 180 mg/day, and this abrupt increase of oxycodone caused stercoral diarrhea. Finally, total blockage of stool developed, resulting in acute abdomen.

## 1. Introduction

Opioids are used for successful cancer pain management and adequate analgesia without excessive adverse effects in patients [1]. The availability and consumption of opioids have increased, and opioids other than morphine, such as oxycodone, have become more widely used. These drugs affect three types of the opioid receptor and subtypes of *μ* receptors differently [2, 3]. Therefore, individual variations in response to opioids must be considered in the management of cancer pain. Oxycodone and morphine have distinctly different pharmacological profiles; however, constipation during oxycodone treatment also occurs like in morphine treatment [4].

## 2. Case Report

A 54-year-old man was referred to the emergency department for severe abdominal pain and diarrhea. He had undergone radiotherapy for squamous cell carcinoma of the external auditory canal six months before; however, he underwent additional radiotherapy again for recurrence of disease. He was otherwise healthy and with no history of abdominal diseases. He had taken oxycodone 10 mg daily for moderate to severe cancer pain in the ear. Initially, he did not complain of constipation, and his cancer pain was well controlled. However, the pain gradually increased over a period of several months, and the dose of oxycodone was increased to 80 mg. His ear pain then worsened and he suddenly became constipated. His dose of oxycodone was then increased to 100 mg, and he was given magnesium hydrochloride for the constipation. However, he self-medicated with oxycodone at dosages of up to 180 mg/day.

**Figure 1:**
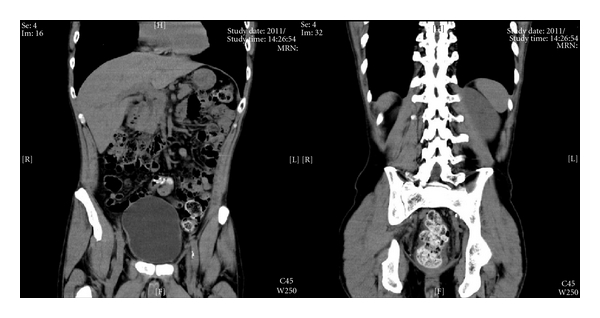
Coronal computed tomography scan showing the amount of colon gas and a large stool mass blocking the rectum.

After five days, he suddenly complained of diarrhea, hence, the laxative was stopped. However, his diarrhea worsened, and acute, severe abdominal pain occurred 6 days after the development of diarrhea, and then he visited the emergency department. An abdominal exam showed a flat, slightly hard abdomen with normal bowel sounds. He showed tenderness in the mid-lower abdomen, but no rebound tenderness. Digital examination of the rectum was performed, and hard stool was detected. Abdominal radiography showed a large amount of gas in the colon, and a computed tomography scan also showed colonic gas and a large stool mass blocking the rectum (Figure 1). This patient was treated with hydration and stool extraction, and the abdominal pain gradually ceased. His pain is now well controlled by percutaneous fentanyl administration.

## 3. Discussion

As mentioned above, oxycodone and morphine have distinctly different pharmacological profiles, however, constipation also occurs in both treatments [2, 3]. Thus, it is necessary to watch out and treat constipation in patients taking opioids [4–7]. 

Until now, acute abdomen caused by opioids has never been reported, although ileus is sometimes seen in postoperative use of opioids [5]. Acute colonic pseudoobstruction after colonoscopy in a patient taking low-dose oxycodone has been reported [8]. Our patient, however, did not undergo colonoscopy. The mechanism of the acute abdominal pain after oxycodone intake in our patient is hypothesized as follows: depending on the increase in oxycodone dosage, slight constipation gradually occurred. After the administration of laxatives, watery intestinal content was being passed as stool; that is, stercoral diarrhea had developed. Finally, total blockage of stool developed, resulting in acute abdominal pain. The abrupt increase in the dosage of oxycodone to 180 mg may have caused this episode.

At first, the oral intake of oxycodone had not caused constipation in this patient. In addition, this patient showed stercoral diarrhea and this made it difficult to make precise diagnosis. The first step to accurate diagnosis in such a situation is to obtain careful medical history. If the patient with acute abdomen took opioids, especially with abrupt increase of opioids, clinicians should think about side effect of the opioid although he complained of diarrhea. Diagnosis for this condition comprises of digital examination and X-rays. After the diagnosis, general treatment for ileus and constipation is needed. Adequate hydration, laxative, and pain relief excepting opioids are essential. In addition to this, an enema and digital stool extraction are applied. In the present case, stool extraction by digital and enema was done under sufficient hydration.

The primary management to avoid the acute abdomen caused by opioids is an adequate control of the opioids as well as laxative [5]. This is important not only for clinicians but also for patients. The second step is opioid switch. Fortunately, this patient's pain is currently well controlled by a fentanyl patch, which has fewer side effects related to the bowels [9, 10]. Appropriate switching of opioids as a clinical maneuver not only may improve medication tolerance but also may avoid adverse side effects.

## 4. Conclusion

Oxycodone is used for successful cancer pain management and adequate analgesia without excessive adverse effects. However, constipation during its treatment may occur and inappropriate use of oxycodone develops abdominal emergency like our case. It is necessary to watch out and treat constipation in patients taking opioids.

##  Conflict of Interests

The authors declare that they have no conflict of interests, any grant, or financial profit related with this study.
